# Use of poly(ε‐caprolactone)‐based films for equilibrium‐modified atmosphere packaging to extend the postharvest shelf life of garland chrysanthemum

**DOI:** 10.1002/fsn3.909

**Published:** 2019-05-01

**Authors:** Peifang Cheng, Xueyan Yun, Chang Xu, Yang Yang, Yumei Han, Tungalag Dong

**Affiliations:** ^1^ College of Food Science and Engineering Inner Mongolia Agricultural University Hohhot China

**Keywords:** equilibrium‐modified atmosphere packaging materials, garland chrysanthemum, gas permeability, poly(propylene carbonate), poly(ε‐caprolactone), shelf life

## Abstract

A uniaxial‐stretched poly(ε‐caprolactone)/poly(propylene carbonate; PCL/PPC) composite film was prepared using a twin‐screw extruder, and its utility as an equilibrium‐modified atmosphere packaging (EMAP) film extending the shelf life of garland chrysanthemums stored at 2~4°C was explored. The oxygen, carbon dioxide, and water vapor penetration properties, mechanical properties, and gas permselectivity of PCL/PPC film used to package garland chrysanthemums were determined and compared to those of controlled low‐density polyethylene (LDPE) and PCL films. Physicochemical properties such as package headspace gas composition, weight loss, leaf color, total chlorophyll content, ascorbic acid content, lipid peroxidation extent, and the sensory traits of garland chrysanthemums were investigated over a storage period of 14 days to compare the preservative effects of the various packages. PPC blending decreased the PCL gas and water vapor permeability and slightly increased the CO_2_ permselectivity. These effects on gas and water vapor permeability, combined with the effects on gas permselectivity, enhanced preservation of packed garland chrysanthemums. Furthermore, an O_2_ inner atmosphere level of 2%~5%, and a CO_2_ concentration not greater than 8%, was established by the PCL/PPC film in the absence of condensation. The results thus suggest that biodegradable film can be used as an EMAP film to better maintain the quality of freshly harvested garland chrysanthemums and to afford a longer shelf life during cold storage compared to LDPE film. Sensory evaluation indicated that the garland chrysanthemums were market‐acceptable after 14 days of storage; LDPE‐packed chrysanthemums were acceptable only up to 8 days of storage. The film thus improved storage life compared to that afforded by LDPE.

**Abbreviations**
ANOVA: analysis of variance
AsA: ascorbic acid
CDTR: carbon dioxide transmission rate
CO_2_: carbon dioxide
EMAP: equilibrium‐modified atmosphere packaging
*E*: Young's modulus
LDPE: low‐density polyethylene
MAP: modified atmosphere packaging
MDA: malondialdehyde
O_2_: oxygen
OTR: oxygen transmission rate
PCL: poly(ε‐caprolactone)
PLW: physiological loss in weight
PPC: poly(propylene carbonate)
RH: relative humidity
WVTR: water vapor transmission rate
α: CO_2_/O_2_ permselectivity
*ε*_*b*_: elongation at break
*σ*_*m*_: tensile strength

## 1 INTRODUCTION

Fresh leafy vegetables constitute an important source of fiber, minerals, and bioactive compounds, such as vitamin C, vitamin A, and polyphones (Baranowski & Ferdyn‐Grygierek, 2011; Kader, 2008; Martínezromero et al., 2007; Pandrangi & Laborde, 2004). However, leafy vegetables are characterized by a relatively limited postharvest shelf life because of deterioration, such as leaf withering combined with yellowing or leaf decay, which is primarily caused by high moisture levels and degradation after harvesting. Demand for the garland chrysanthemum is increasing both because of its fragrance and its abundant bioactive constituents, including carotene, vitamin C, volatile essential oils, and choline, which are regarded as health‐promoting components (Flamini, Cioni, & Morelli, 2003).

In recent years, many different methods and polymeric films have been developed to package leafy vegetables, to extend shelf life, inhibit senescence, maintain quality (i.e., color, flavor, nutritional components, and texture), and inhibit rot. Various preservatives, including regulators of physiological activity and ethylene inhibitors, are often used to these ends. Gibberellins, combined with appropriate storage conditions, greatly reduce protein loss and catalase activity, retarding parsley senescence (Lers, Jiang, Lomaniec, & Aharoni, 2008). 1‐methylcyclopropene (1‐MCP; an ethylene inhibitor) can be used to significantly extend the shelf life of kale borecole, reducing yellowing and retarding chlorophyll degradation at 5°C, thus ultimately maintaining sensorial quality during storage (Cefola, Amodio, Rinaldi, Vanadia, & Colelli, 2010).

Recent studies have focused on combining modified atmosphere packaging (MAP) techniques using different films to improve the shelf life and safety of many types of fresh fruits and vegetables (Cliffebyrnes & O'Beirne, 2005; Fernández, Aspe, & Roeckel, 2009; Fernández‐León et al., 2013; Irtwange, 2006; Severino et al., 2015; Simón, González‐Fandos, & Vázquez, 2010). For many years, equilibrium‐modified atmosphere packaging (EMAP) has been conveniently used to package fresh fruits and vegetables, effectively prolonging shelf life by reducing weight loss, retarding softness, and retaining flavor (Almenar et al., 2007; Jacxsens, Devlieghere, & Debevere, 2002; Mistriotis, Briassoulis, Giannoulis, & D'Aquino, 2016). Synthetic petroleum‐based polymeric films, varying in terms of permeability to O_2_, CO_2_, and water vapor, have been widely used in combination with cool storage to delay senescence and extend shelf life (Del Nobile, Licciardello, Scrocco, Muratore, & Zappa, 2007; Mangaraj, Goswami, & Mahajan, 2009); polyethylene (PE) and polyvinyl chloride (PVC) films have found many applications in retail and wholesale packaging (Exama, Arul, Lencki, Lee, & Toupin, 1994). However, these films are nonbiodegradable and nonrenewable; selection of an appropriate packaging film is crucial for modifying atmospheric O_2_ and CO_2_ concentrations to extend shelf life. Furthermore, the levels of CO_2_ and O_2_ transmitted through packaging material must be optimal for products subjected to EMAP. For example, postharvest, fresh‐cut green bell pepper (*Capsicum annuum* L.) can be maintained in good condition for up to 9 days when exposed to CO_2_ levels of 7% combined with O_2_ levels of 13%–14%, maintaining optimum microbiological quality with no off‐odor (Ranjitha, Rao, Shivashankara, & Roy, 2015). Given the environmental problems caused by the increased use of traditional petroleum‐based plastic films (Tharanathan, 2003), the use of degradable food packaging materials is attracting increasing interest. Biopolymers, including poly(l‐lactide; PLLA)‐, poly(ε‐caprolactone; PCL)‐, and polypropylene carbonate (PPC)‐based materials, have been investigated in the context of packaging; these materials are naturally biodegradable (Auras, Harte, & Selke, 2004; Dong, Yu, et al., 2015; Dong, Yun et al., 2015; Yun et al., 2017), which can be quantitatively degraded by action of microorganisms into some other nontoxic small molecules such as CO_2_ and H_2_O under aerobic or anaerobic conditions (Chiellini & Solaro, 2004). Thus, they are perceived as environmentally friendly materials and interestingly better suited for a number of applications such as shopping bags, food service packaging materials, and agricultural mulch films (Kasirajan & Ngouajio, 2013). For example, the quality of tomatoes stored in biodegradable bags was comparable to that of tomatoes stored in low‐density polyethylene (LDPE) bags over 21 days (Kantola & Helen, 2010). In addition, microbial and physicochemical properties, such as color, firmness, and the ascorbic acid level, of peppers packaged in PLLA‐based biodegradable films, were similar to those packaged in LDPE film. Given the lower water vapor transmission rate of LDPE films, more molds and yeasts were observed on peppers packed with LDPE than biodegradable film (Koide & Shi, 2007).

Poly(ε‐caprolactone; PCL) is an aliphatic polyester composed of hexanoate repeat units (Labet & Thielemans, 2009). PCL effectively maintains the sensory and physicochemical features of foods, as observed during cold storage of fresh strawberries (Yun et al., 2017). PPC is an aliphatic carbonate polyol produced via catalytic copolymerization of carbon dioxide and propylene oxide (Barreto, Hansen, & Fredriksen, 2012), and is a potentially useful food packaging material given its excellent barrier properties, ready availability, and low cost (Darensbourg et al., 2009). However, because of the poor mechanical properties of the material and the low glass transition temperature, applications to food packaging have been limited (Koning et al., 2001; Qin & Wang, 2010). Therefore, biodegradable PCL/PPC‐blended films might find ready applications in EMAP of fresh produce.

However, few data on applications of such eco‐friendly packaging materials to enhance garland chrysanthemum shelf life are available. We thus explored the utility of biodegradable‐based films exhibiting appropriate gas permeability. We determined whether PCL/PPC‐blended films exhibiting suitable gas permeability and CO_2_ permselectivity would prolong the shelf life of garland chrysanthemums during cold storage.

## 2 MATERIALS AND METHODS

### 2.1 Materials and sample preparation

PCL (*M*_n_ = 1.3 × 10^6^) was purchased from Esun Advanced Materials Co. Ltd. (Shenzhen, China). PPC was provided by Chemical Materials Co. Ltd. (Nanyang, China; Yun et al., 2017). PCL and PCL/PPC‐blended (60/40 wt%) films of thickness 40 μm were prepared using a twin‐screw extruder system (PPT‐3/SJ2‐20‐250; Guangzhou POTOP Experimental Analysis Instrument Co. Ltd., Guangzhou, China). LDPE bags of similar thickness were purchased from Packing Product Co. Ltd. (Shenzhen, China). Each bag featured an area of 620 cm^2^ available for gas exchange. The bags were 31 cm × 20 cm in dimensions and were manufactured using a heat‐sealing machine (DBF‐900; Wenzhou Dingli Package Machine Manufacture Co. Ltd., Wenzhou, China).

### 2.2 Sample packaging and storage

Garland chrysanthemums (*Chrysanthemum coronarium* L.) were obtained from our local agricultural cooperative at the end of February and directly transported by car to the laboratory in corrugated cases. The produce was sorted to eliminate mechanically damaged, overaged, and tender fruit; we selected fruits of uniform size and color. After precooling at 2~4°C for 2 hr, samples were randomly selected and weighed (about 150 g/sample), heat‐sealed in prepared bags, and stored at 2~4°C and 66% relative humidity (RH) in a cabinet that contained no other fruits/vegetables (KGES‐1200 standard). The package headspace volume was about 116 cm^3^/bag. Physicochemical and sensory analyses were performed every 2 days. Each packing film was evaluated three times.

### 2.3 Gas permeability

The oxygen transmission rate (OTR) and carbon dioxide transmission rate (CTR) of PCL, PCL/PPC, and LDPE films were measured in duplicate at 4°C using a manometric gas permeability tester (Lyssy L100‐5000; Systech Instruments, Oxford, UK) employing the ASTM 1434‐82 standard.

### 2.4 Water vapor permeability

The water vapor transmission rate (WVTR) of 1‐cm^2^ disks of PCL, PCL/PPC, and LDPE films was determined at 25°C and 65% RH using a Permatran‐W Model 3/61 water vapor permeability meter (Mocon Inc., Minneapolis, MN, USA) employing the ASTM E96 default method.

### 2.5 Mechanical properties

A Mecmesin MultiTest XH‐8750 universal tensile machine (Xinghui Electronic Co. Ltd., Dongguan, China) equipped with a 100‐N static load cell was used to measure tensile strength (*σ*_*m*_), elongation at breakage (*ε*_*b*_), and the Young's modulus (*E*) of all films using the ASTM D882‐09 standard method (Wang et al., 2015). The films were conditioned at room temperature for at least 1 week prior to testing. Film samples were cut into dumbbell‐shaped strips using an engraving tool. Each sample was evaluated at least six times. Mechanical parameters were calculated by plotting stress–strain curves.

### 2.6 Gas composition of the headspace

The O_2_ and CO_2_ concentrations in the headspace were monitored during storage using a headspace O_2_/CO_2_ analyzer (Model 6600; Systech Instruments). Just before measurement, the packages were removed from storage and silicone septa were stuck to the film surfaces. Then, needles attached to the analyzer were inserted into the packages. The instrument was calibrated by reference to air before use. The gas composition of each package was measured, and the results are reported as expected percentages of air composition.

### 2.7 Sensory evaluations and marketable quality testing

Sensory quality was evaluated by a trained, 10‐person panel (Tudela et al., 2013). The organoleptic evaluation indicator were visual appearance, texture, general acceptability, and smell. Visual quality was scored using a nine‐point scale in terms of color, leaf firmness, and glossiness, where 9 = excellent (full freshness), 5.5 = threshold of marketability, and 1 = inedible (serious withering, yellowing, or decay). Off‐odor was evaluated using a five‐point scale, where 5 = severe decomposition (dense abnormal taste), 3 = moderate (no scent and no undesirable odor), and 1 = dense fragrance (no off‐odor). Three groups of samples were evaluated every 2 days after packaging. Chamber performance was evaluated in terms of shelf life by the number of days over which the sensory score was maintained at ≥5.5.

### 2.8 Physicochemical analyses

#### 2.8.1 Physiological loss in weight (PLW)

The weight of each package was measured on day 0, and on sampling days, using an Adventurer precision balance (PRECISA JA‐5003B). The PLW was the percentage loss of initial weight.

#### 2.8.2 Ascorbic acid concentration

The ascorbic acid content was determined spectrophotometrically. Fresh garland chrysanthemums (10 g) were weighed and homogenized in 50 g/L trichloroacetic acid (TCA; 20 ml); supernatant samples (1 ml) were added to 50 g/L TCA (1 ml) and immediately subjected to absorbance measurements at 534 nm. Each sample was tested three times. The results are expressed in mg/100 g fresh weight (FW).

#### 2.8.3 Leaf color

Surface color was measured with a colorimeter (TCP2‐A; Beijing Xinaoyike Photoelectric, Beijing, China) immediately after opening each bag. The color values were expressed as L*, a*, and b* values as recommended by the Commission International de L'Eclairage (CIE). The numerical values of a* and b* were directly converted into b*/a* values, and leaf color was evaluated in terms of the Hunter laboratory hue angle [tan^−1^ (b*/a*)] (Zorić, Pedisić, Kovačević, Ježek, & Dragović‐Uzelac, 2016).

#### 2.8.4 Total chlorophyll content

Chlorophyll content was determined spectrophotometrically by measuring the absorbance of extracted leaf liquids at appropriate wavelengths. Garland chrysanthemum leaves (1 g) were extracted into 3 ml 80% (v/v) acetone after homogenization using silica sand and calcium carbonate powder at room temperature. The absorbances of all samples were measured at 645 and 663 nm (detecting chlorophyll b and chlorophyll a, respectively), as described by Garrido, Tudela, Hernández, and Gil (2016). The results are expressed in mg chlorophyll/g FW.

#### 2.8.5 Lipid peroxidation

The extent of lipid peroxidation was measured by assaying tissue malondialdehyde (MDA) content, as described by Hernández, Rubio, Olmos, Ros‐Barceló, and Martínez‐Gómez (2004). Fresh samples (1.0 g) were homogenized in 100 g/L TCA (5 ml) and centrifuged at 10,000 *g* for 20 min. The supernatants (2.0 ml) were mixed with 0.67% (w/v) TBA diluted in 100 g/L of TCA and incubated at 95°C for 30 min; the reactions were then stopped by placing the tubes in an ice‐water bath. The absorbance of supernatants at 532 nm was corrected for nonspecific absorbance by subtracting the absorbance at 450 and 600 nm. Four samples from two replicates (two samples per replicate) were measured at each sampled storage time. The results are expressed in nmol MDA/g FW.

### 2.9 Data analysis

One‐way analysis of variance (ANOVA) was used to compare physicochemical and sensorial data using SPSS software (ver. 20.0; IBM Corp., Armonk, NY, USA). The average values were compared using the Turkey B test. Least significant differences (LSDs, *p *< 0.05) at a confidence interval of 95% were calculated. Data are expressed as means ± standard deviation.

## 3 RESULTS AND DISCUSSION

### 3.1 Film permeability

The gas permeability of packaging materials is vital to the postharvest shelf life of fresh produce. We explored the gas permeability and CO_2_/O_2_ permselectivity of PCL, PCL/PPC, and LDPE films. The OTRs, CTRs, water vapor permeability, and CO_2_:O_2_ permeability ratios of all films are listed in Table 1. The OTRs and CTRs of PCL/PPC‐blended films were decreased with the addition of PPC, whereas the LDPE film exhibited higher OTRs and CTRs compared with the other two films.

**Table 1:** Values of O_2_, CO_2,_ and water vapor permeability of PCL, PCL/PPC, and LDPE

Packaging film	OTR (m^3^/m^2^·24 hr)	CTR (ml/m^2^·24 hr)	WVTR (g/m^2^·24 hr)	Permselectivity
PCL	485.7 ± 6.0^b^	5,011 ± 110^b^	783 ± 5^a^	10.3^ab^
PCL/PPC	209.2 ± 5.1^bc^	2,260 ± 152^c^	466 ± 6^b^	10.8^a^
LDPE	2,745 ± 170^a^	8,752 ± 161^a^	5.8 ± 0.5^c^	3.2^c^

Values are mean ± standard deviation of triplicate determinations. Means on the same column with different sets of superscripts are statistically different (*p* ≤ 0.05).

As shown in Table 1, the CTRs of PCL/PPC‐blended films decreased significantly (*p *˂ 0.05) with the addition of PPC,but regarding the OTRs, no significant difference was observed between the PCL and PCL/PPC films (*p* > 0.05). For example, the OTRs and CTRs of PCL/PPC film were 56.9% and 54.9% lower than those of PCL film, reflecting the excellent gas barrier performance of PPC (Dong et al., 2014). Also, the PCL/PPC film exhibited slightly higher CO_2_/O_2_ permselectivity (Table 1; defined as the CTR:OTR ratio) compared with PCL film; this is an important parameter of EMAP films. CO_2_/O_2_ permselectivity determines the O_2_/CO_2_ concentration inside packaging that meets the respiratory needs of fruits and vegetables. Suitable EMAP system ratios can be obtained by altering film gas permselectivity (Alati & Hotchkiss, 2003).

In general, EMAP packaging materials with gas selectivity (CO_2_:O_2_ permeability ratio) of about 8~10 are optimal for preservation of fresh produce with high respiration rates (Hayakawa, Henig, & Gilbert, 2010; Lee, Haggar, & Yam, 1992). However, most films exhibit relatively low CO_2_:O_2_ permeability ratios (Herrera, Mathew, & Oksman, 2014). PLLA, for example, exhibits a CO_2_:O_2_ permeability ratio of only about 3 (Song et al., 2017; Song et al., 2016). In this work, the gas permselectivity of PCL and PCL/PPC films was 10.3 and 10.8, respectively, much greater than that of LDPE (3.2; Table 1). Based on the comprehensive consideration of gas permeability and gas permselectivity of those films in theory, it would be more beneficial for the PCL/PPC film packaging to the establishment of relatively lower‐O_2_ as well as higher‐CO_2_ equilibrium‐modified atmosphere for storage of fresh products (Lidster & Leung, 2008).

The water vapor transmission rate (WVTR) of PCL/PPC was 466 g/m^2^·24 hr, and that of pure PCL was 783 g/m^2^·24 hr (i.e., about 40.5% less), indicating that blending of PPC suppressed the loss of excessive moisture from the package more so that did PCL. Nevertheless, the WVTR of PCL/PPC was approximately 80‐fold greater than that of the LDPE film (5.8 g/m^2^·24 hr; Table 1); this may be why no condensation formed in PCL/PPC packages. Packaging films with poor water permeability are associated with the development of internal condensation on the surfaces of fresh products, which may encourage mold growth, as seen with LDPE films in previous reports (Koide & Shi, 2007; Srinivasa, Baskaran, Ramesh, Prashanth, & Tharanathan, 2002; Suparlan, 2003).

Therefore, it is reasonable to suggest that PCL/PPC film packaging would be associated with a relatively lower headspace oxygen level than LDPE film packaging, given the differences in O_2_ and CO_2_ permeability and gas selectivity. The higher water vapor permeability of the PCL/PPC film inhibited condensation; this was not the case after LDPE packaging.

### 3.2 Mechanical properties

Strength and toughness are the two most important mechanical property parameters of plastic films. Mechanical properties including tensile strength (*σ*_*m*_), Young's modulus (*E*), and elongation at breakage (*E*_*b*_) of all films are listed in Table 2; Typically, *σ*_*m*_ and *E* as well as *E*_*b*_ are used to characterize the tensile resistance, rigidity, and toughness of packaging materials, respectively. All the data were calculated using stress–strain curves. As shown in Table 2, PCL could undergo large deformation and significant elongation prior to breakage, associated with relatively low *E* and *σ*_*m*_ values. The *E* and *σ*_*m*_ values of PCL/PPC improved to 30.6 and 236.6 MPa, respectively, showed significant difference (*p *˂ 0.05) compared to those of pure PCL film. Furthermore, the elongation at breakage exceeded 246%, reflecting relatively high toughness. In addition, the tensile resistance, rigidity, and toughness were superior than those of the LDPE film. Our preliminary data (data not shown) indicated that mechanical properties presented increased trends with increasing PPC blending ratio from 10% to 40%, however, mechanical properties are severely compromised when the blend percentage of PPC increases to 50%, because of the poor compatibility between PCL and PPC during blending of PCL with PPC. Thus, the PCL/PPC (40 wt%)‐blended films were chosen as the packaging film of garland chrysanthemum.

**Table 2:** Mechanical properties of films conditioned at 50% RH

Sample	*ε*_*b*_ (%)	*σ*_*m*_ (MPa)	*E* (MPa)
PCL	214.1 ± 12.5^ab^	23.2 ± 3.6^b^	113.4.4 ± 5.9^bc^
PCL/PPC	246.3 ± 17.6^a^	30.6 ± 9.8^a^	236.6 ± 35.4^a^
LDPE	161.5 ± 22.5^bc^	19.2 ± 1.7^bc^	155.4 ± 12.8^b^

Values are mean ± standard deviation of triplicate determinations. Means on the same column with different sets of superscripts are statistically different (*p* ≤ 0.05).

### 3.3 Headspace gas composition

**Figure 1:**
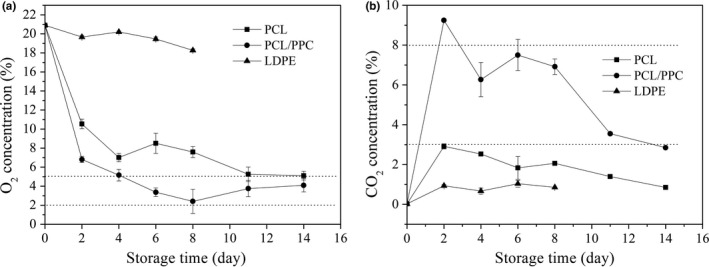
Evolution of the headspace gas composition O_2_ (a) and CO_2_ (b) of the three different package bags with 150 g garland chrysanthemum at 2~4°C. The vertical bars indicate the standard errors of three replicates

Maintenance and adjustment of atmospheric composition are pivotal for fresh produce quality (Hyun & Lee, 2017). Equilibrium‐modified atmospheres developed inside the packaging films, reflecting the gas permeability of the packaging materials and respiratory activity of the garland chrysanthemums. Figure 1 shows the changes in O_2_ (a) and CO_2_ (b) concentrations within packages during storage.

In the case of PCL and PCL/PPC‐blended films, the CO_2_ concentration increased rapidly, and that of O_2_ decreased rapidly during the first 2 days of storage, which principally attributed to a sharp rise in the postharvest respiration of fruits and vegetables at the initial storage time (Lee, Arul, Lencki, & Castaigne, 2010),thus resulting in a rapid depletion of O_2_ as well as a quick accumulation of CO_2_ inside the package. However, the higher CO_2_ in turn inhibited the respiration of garland chrysanthemum after 2 days. In addition, because garland chrysanthemum has a high respiration rate, the modification of atmosphere in the package headspace readily happened according to their different gas permeability and gas permselectivity of the films. When the respiration rate of garland chrysanthemum gradually matched the gas permeability of the film, a stable internal atmosphere would be established. Owing to the relatively low O_2_ permeability of PCL/PPC films compared with PCL, too much O_2_ in air could not penetrate the films, but the redundant CO_2_ in the packages could escape due to its high gas permselectivity. Consequently, a steady state was achieved after 4 days of storage, and the concentration of O_2_ then persisted in the range 2.3%~4.9% as well as CO_2_ concentration of 2.9%~7.3% for PCL/PPC film packages. In contrast, a higher O_2_ levels with the range of 5.1%~8.3% and lower CO_2_ levels of 1.1~2.9 were observed in PCL film package. However, for the LDPE film packages, the gas composition was close to that of air throughout storage.

Too high a CO_2_ level and/or too low an O_2_ concentration can induce anaerobic metabolism, triggering physiological damage or decay (Domínguez, Lafuente, Hernández‐Muñoz, & Gavara, 2016; Watkins, 2000). Overall, high‐level CO_2_ accumulation inhibits aerobic metabolism and may induce off‐odors caused by anaerobic (fermentative) metabolism (Artés, Gómez, & Artéshernández, 2006). EMAP requires adjustment and control of both O_2_ and CO_2_ levels to prevent anaerobic respiration and accumulation of hazardous substances such as ethyl alcohol and acetaldehyde (Giuggioli, Briano, Baudino, & Peano, 2015), which can destroy cell integrity. For the garland chrysanthemum, the recommended optimal gas levels are 2%–5% for O_2_ and not greater than 8% for CO_2_ (Sandhya, 2010). CO_2_ levels above 12% exerted negative effects on flavor, firmness, and the acid content of Duke blueberries, although high CO_2_ levels were required to inhibit *Botrytis cinerea* metabolism (Harb & Streif, 2004). The effects of film permeability on O_2_ and CO_2_ concentrations are as important as other factors, such as storage temperature, respiration rate, film surface area, void volume, and sample weight (Fonseca, Oliveira, & Brecht, 2002). We found that the O_2_ and CO_2_ levels in PCL/PPC film packages were consistently within acceptable limits, usefully preserving garland chrysanthemums.

### 3.4 Sensory quality

**Figure 2:**
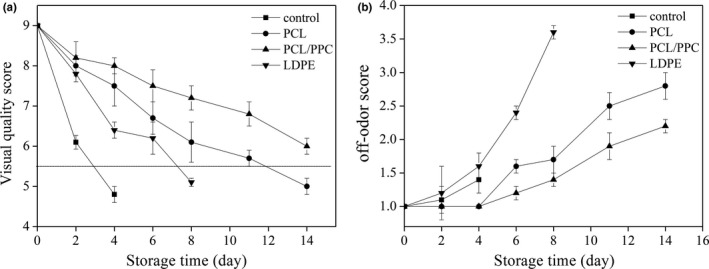
The sensory quality scores in visual quality (a) and off‐odor (b) of garland chrysanthemum packaged in different package bags and stored at 2~4°C for up to 14 days. The vertical bars indicate the standard errors of three replicates

**Figure 3:**
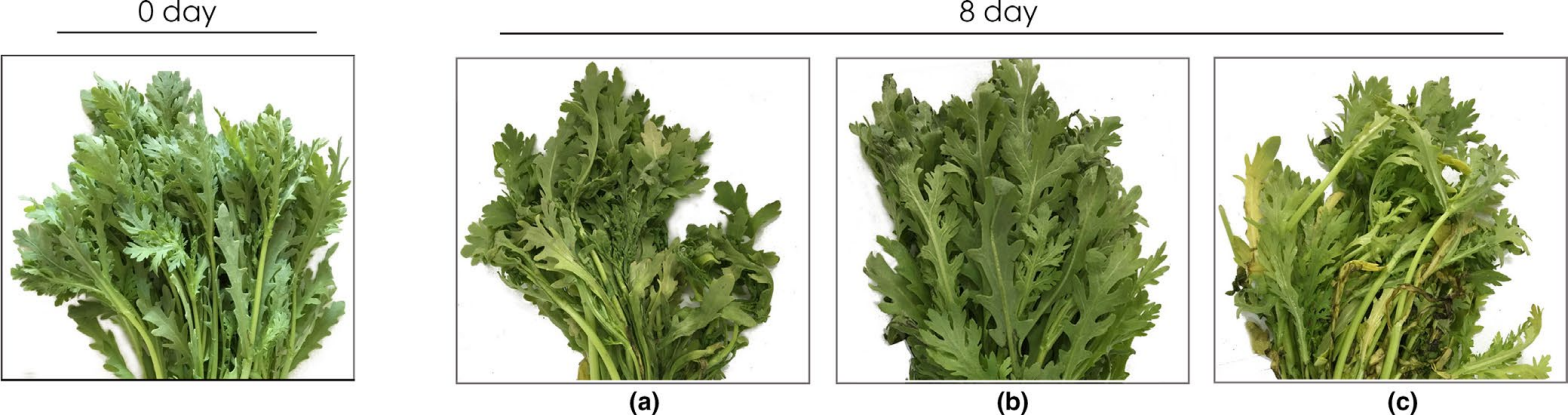
Image of garland chrysanthemum removed from the package PCL (a), PCL/PPC (b), and LDPE (c) package bags at 0 and 8th days and stored at 2~4°C

**Figure 4:**
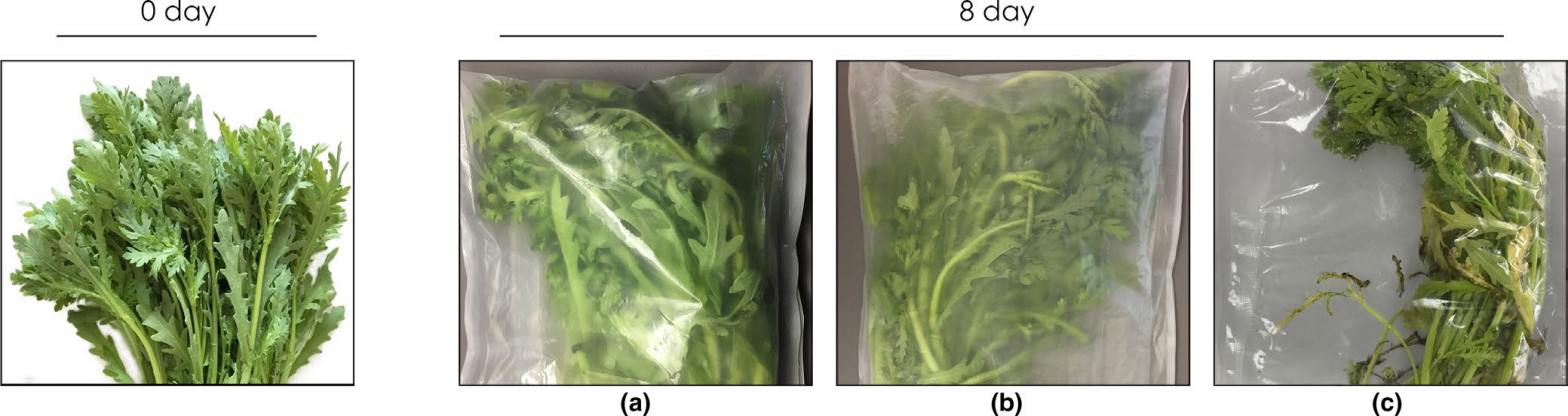
Image of garland chrysanthemum packaged in PCL (a), PCL/PPC (b), and LDPE (c) at 0 and 8th days stored at 2~4°C

Sensory quality and marketability were subjectively evaluated by a trained panel of 10 individuals who scored both visual appearance and off‐odor (Figure 2a,b). The packaging pouches of, and chrysanthemums stored in, different packaging materials for 8 days are shown in Figures 3 and 4, respectively. The highest mean score (7.2 ± 0.3) for marketability was associated with the PCL/PPC treatment, as were maintenance of green color and leaf stiffness after 8 days of storage (Figure 3). The scores and images indicate that no significant changes in visual appearance were evident in garland chrysanthemums packed in PCL and PCL/PPC films. However, significant differences (mean score of 5.1 ± 0.1, below the limit of marketable acceptance) were observed after LDPE treatment. After 8 days of refrigerated storage, visible rotting and yellowing of almost 67% of samples were evident after LDPE packaging (Figure 3). Little water was apparent in LDPE packages, or on the surfaces of chrysanthemums, although retention of leaf firmness was only partial. No condensation was evident after PCL or PCL/PPC packaging; PCL‐based films affording better water vapor transmission allow for timely water permeation (Figure 4).

In terms of odor changes, the PCL/PPC treatments scored lower than the LDPE treatment after 4 days of storage; the PCL and PCL/PPC treatment scores did not differ. However, after storage for 8 days, a slight off‐odor developed in LDPE treatment samples; the scores differed significantly (*p *< 0.05) from those of other treatments. These results are similar to those observed on EMAP of baby spinach under high CO_2_ and low O_2_ pressures (Garrido et al., 2016). LDPE packages generated higher O_2_ and lower CO_2_ concentrations than the other packages, because of poor gas barrier properties that accelerated tissue senescence and lipid peroxidation, ultimately leading to the development of off‐odors (in agreement with the findings of previous studies; Garrido et al., 2016; Srinivasa et al., 2002).

### 3.5 Physicochemical analyses of garland chrysanthemum

#### 3.5.1 Weight loss

**Figure 5:**
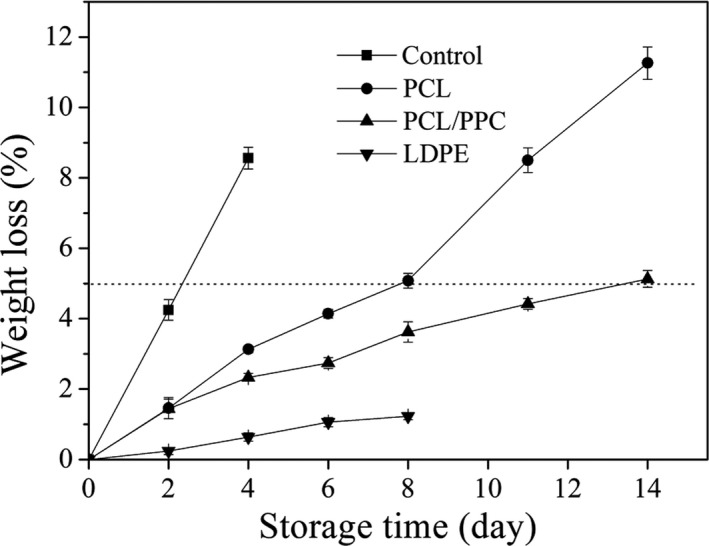
Change in weight of garland chrysanthemum packaged in different package bags and stored at 2~4°C for up to 14 days. The vertical bars indicate the standard errors of three replicates

The water contents of leafy vegetables are generally above 90%, maintaining physiological activity and retaining freshness (such as leaf stiffness). Water loss via transpiration causes leaf withering and shriveling. Weight loss during storage at 2~4°C after packing using the three films is shown in Figure 5. A significant difference was apparent among LDPE‐, PCL‐, and PCL/PPC‐packaged fruits after 4 days of storage (*p *< 0.05). The high water vapor barrier of the LDPE film caused this difference. Freshness loss is generally associated with 3%~10% weight loss (Ben, 1987). Garland chrysanthemums packed in PCL/PPC film and stored at 2~4°C remained marketable after 14 days of storage. Although LDPE packaging was associated with low‐level weight loss, condensation favored mold growth, as shown in the sensory evaluation. No condensate formed in PCL or PCL/PPC packages.

#### 3.5.2 Ascorbic acid content

**Figure 6:**
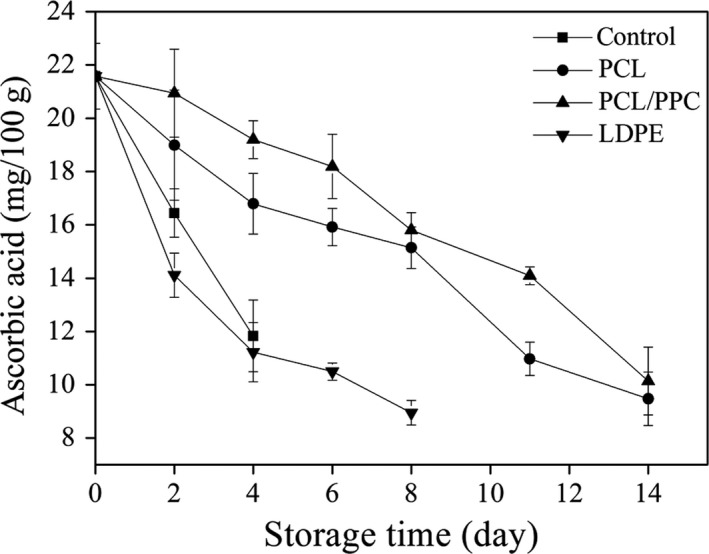
Change in ascorbic acid (mg/100 g) of garland chrysanthemum packaged in different package bags and stored at 2~4°C for up to 14 days. The vertical bars indicate the standard errors of three replicates

Ascorbic acid levels are high in many leafy vegetables; ascorbic acid is an important nutritional resource, serving as a cofactor in many enzymatic reactions (Niklis, Siomos, & Sfakiotakis, 2002). Changes in ascorbic acid content during storage are shown in Figure 6. In general, ascorbic acid content decreased gradually during storage in all packages, presumably because all samples were placed in cold storage on day 1 (Hu, Fang, Yang, Ma, & Zhao, 2011).

Relative to the initial values, the ascorbic contents of the control and LDPE groups decreased dramatically, by 45% and 48%, after 4 days. In the PCL/PPC and PCL groups, the ascorbic acid contents were significantly higher than those of the LDPE and control groups over the first 8 days (*p *< 0.05). The ascorbic acid contents of PCL/PPC‐packaged products were higher than those of the other two groups during storage. A low O_2_ level in the bag preserves the ascorbic acid level. The O_2_ concentrations in PCL/PPC packages were lower than those in PCL and LDPE packages. Moreover, relatively higher CO_2_ concentrations developed in PCL/PPC‐packaged products. CO_2_ can form hydrogen bonds with polar groups in cell walls, thus preventing the expression of certain enzymes and retarding ascorbic acid decreases (Jiang, Tian, & Xu, 2002; Qian, Bai, Xin, Cai, & Xiao, 2009); this presumably occurred in the present study.

#### 3.5.3 Changes in leaf color

**Figure 7:**
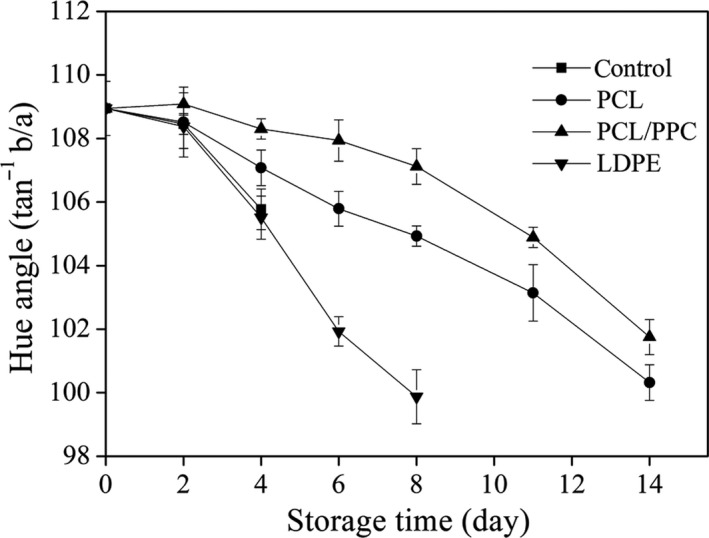
Change in hue angle of garland chrysanthemum packaged in different package bags and stored at 2~4°C for up to 14 days. The vertical bars indicate the standard errors of three replicates

**Figure 8:**
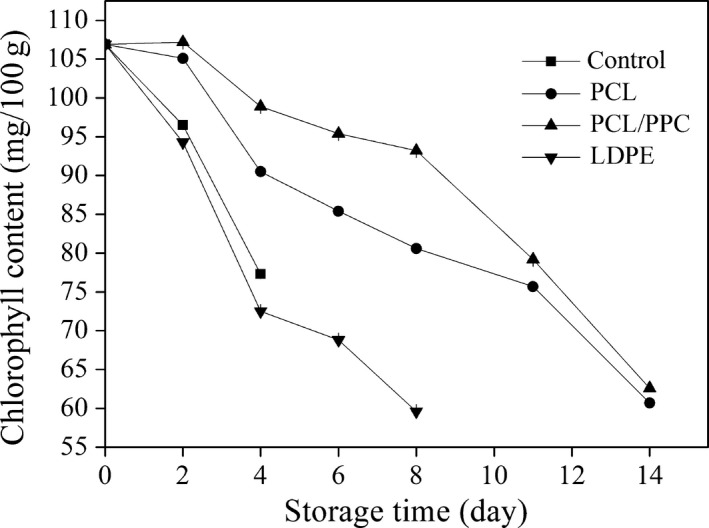
Change in chlorophyll content of garland chrysanthemum packaged in different package bags and stored at 2~4°C for up to 14 days. The vertical bars indicate the standard errors of three replicates

Changes in leaf color and yellowing are used to evaluate the esthetic quality of green leafy vegetables, and their postharvest maturity, as well as senescence developing during storage (Oboh & Akindahunsi, 2004). Able, Wong, Prasad, & O'Hare (2002) showed that the lower the hue angle, the greater the extent of leaf yellowing (positive a* value and negative b* value). Variations in hue angle by treatment are shown in Figure 7. PCL/PPC treatment ensured remarkably higher (*p *< 0.05) preservation of green color on day 8 compared to LDPE treatment (Figure 3). The hue angle and total chlorophyll level (Figure 8) reflect the extent of chlorophyll degradation and retention of green color after PCL/PPC treatment throughout storage. Color changes are attributable to micromodification of the atmosphere within packaging films; these approach the recommended gas proportion for garland chrysanthemums, thus retarding chlorophyll degradation to carotene (pale yellow) and hydroxylated carotenoids (yellow).

#### 3.5.4 Total chlorophyll content

Chlorophyll is the chief source of green color in leafy vegetables and is the organoleptic indicator most valued by consumers. PCL/PPC film delayed the reductions in chlorophyll levels from the initial values by retarding chlorophyll degradation. In contrast, the total chlorophyll content fell by 44.2% from the initial level after packaging in LDPE over 8 days of storage (Figure 8).

Chlorophyll loss is reduced in vegetables stored at higher CO_2_ levels (Shewfelt, Batal, & Heaton, 1984). Several studies have found that the total chlorophyll content of leafy greens, including broccoli, chives, garden peas, and asparagus, is better retained at low and relatively high levels of CO_2_ (Imahori et al., 2004; Jamie & Saltveit, 2002; Pariasca, Miyazaki, Hisaka, Nakagawa, & Sato, 2000; Tenorio, Villanueva, & Sagardoy, 2004). Thus, the ambient atmosphere inhibits chlorophyllase and/or peroxidase activities, reducing chlorophyll degradation. As mentioned above, an atmospheric composition of 2%~5% O_2_ and 8% CO_2_ for garland chrysanthemums packed in PCL/PPC bags over 14 days of storage retarded the changes associated with leaf senescence, such as chlorophyll degradation.

#### 3.5.5 Lipid peroxidation

**Figure 9:**
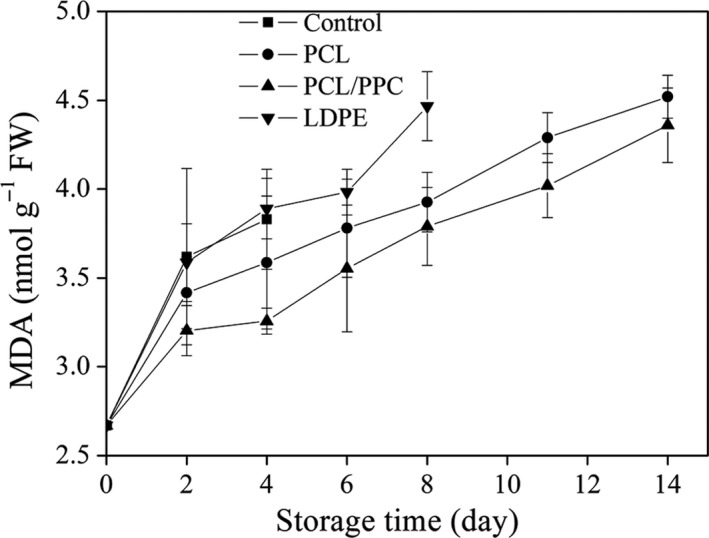
Change in malondialdehyde (MDA) content of garland chrysanthemum packaged in different package bags and stored at 2~4°C for up to 14 days. The vertical bars indicate the standard errors of three replicates

Malondialdehyde has been suggested to be the major chemical responsible for cell membrane lipid peroxidation of plant tissue. The extent of lipid peroxidation of fruits and vegetables can be determined by measuring tissue MDA levels. The extent of lipid peroxidation of garland chrysanthemums packaged in biodegradable films was less than that of samples packaged in LDPE to day 4 of storage (Figure 9; reductions of 14.9% and 4.7%, respectively, for the PCL/PPC and PCL samples). Several studies have found that MDA content was significantly affected by atmospheric composition (Xing et al., 2010).

Equilibrium‐modified atmosphere packaging, a technically mature packaging strategy for preservation of fresh produce, effectively delays membrane lipid degradation, preserving the integrity of cell membranes (Wang, Tian, & Xu, 2005). Any increase in lipid peroxidation yielding MDA disrupts cell membranes, accelerating leaf senescence. Baby spinach stored under low‐ and high‐pressure CO_2_ exhibited less MDA accumulation than leaves stored under moderate and high CO_2_ pressure (Garrido et al., 2016). Therefore, the increased MDA content evident after LDPE treatment can be explained by oxidative damage to the cell membrane, caused by the relatively high O_2_ and low CO_2_ levels. Thus, our biodegradable films inhibit membrane lipid peroxidation over 14 days of storage and thus better maintain the integrity of garland chrysanthemum leaf tissue.

## 4 CONCLUSION

Senescence and decay of garland chrysanthemums were inhibited on packaging with PCL/PPC film followed by storage at 2~4°C; the film afforded appropriate gas and water vapor permeabilities. Relatively constant gas conditions developed spontaneously, with maintenance of leaf firmness and inhibition of physiological and biochemical reactions, compared to the control group. In summary, PCL/PPC bags were useful for commercial postharvest packaging of garland chrysanthemums, maintaining product quality and extending the shelf life for up to 14 days, as well as reducing packaging waste.

## CONFLICT OF INTEREST

The authors have declared no conflict of interest.

## ETHICAL REVIEW

This study does not involve any human or animal testing.

## AUTHORS’ CONTRIBUTIONS

P. F. Cheng conducted most of the experiments, completed statistical analysis, interpreted results, and edited the manuscript. X. Y. Yun and C. Xu completed property testing and analysis of the packaging materials. Y. Yang conducted the organoleptic evaluation process of the garland chrysanthemum. Y. M. Han provided some guidance on the preservation experiment design of garland chrysanthemum. T. Dong designed the study, optimized the preparation process of the package film, supervised data collection and processing, and revised the manuscript.

## References

[fsn3909-bib-0001] Able, A. J. , Wong, L. S. , Prasad, A. , & O'Hare, T. J. (2002). 1‐MCP is more effective on a floral brassica (*Brassica oleracea* var. italica L.) than a leafy brassica (*Brassica rapa* var. chinensis). Postharvest Biology & Technology, 26(2), 147–155. 10.1016/S0925-5214(02)00011-X

[fsn3909-bib-0002] Alati, T. , & Hotchkiss, J. H. (2003). The role of packaging film permselectivity in modified atmosphere packaging. Journal of Agricultural and Food Chemistry, 51(14), 4133–4138. 10.1021/jf034191b 12822958

[fsn3909-bib-0003] Almenar, E. , Del‐Valle, V. , Hernández‐Muñoz, P. , Lagarón, J. M. , Catalá, R. , & Gavara, R. (2007). Equilibrium modified atmosphere packaging of wild strawberries. Journal of the Science of Food & Agriculture, 87(10), 1931–1939. 10.1002/(ISSN)1097-0010

[fsn3909-bib-0004] Artés, F. , Gómez, P. A. , & Artéshernández, F. (2006). Modified atmosphere packaging of fruits and vegetables. Stewart Postharvest Review, 2(5), 1946–13.

[fsn3909-bib-0005] Auras, R. , Harte, B. , & Selke, S. (2004). An overview of polylactides as packaging materials. Macromolecular Bioscience, 4(9), 835–864. 10.1002/(ISSN)1616-5195 15468294

[fsn3909-bib-0006] Baranowski, A. , & Ferdyn‐Grygierek, J. (2011). Numerical analysis of the energy consumption in the office building. Rynek Energii, 2(2), 171–175.

[fsn3909-bib-0007] Barreto, C. , Hansen, E. , & Fredriksen, S. (2012). Novel solventless purification of poly(propylene carbonate): Tailoring the composition and thermal properties of PPC. Polymer Degradation & Stability, 97(6), 893–904. 10.1016/j.polymdegradstab.2012.03.033

[fsn3909-bib-0100] Ben, Y. S. (1987). Transpiration, water stress, and gas exchange In Weichmann J. (Ed.), Postharvest physiology of vegetables (pp. 113–170). New York, NY: Marcel Dekker, Inc.

[fsn3909-bib-0009] Cefola, M. , Amodio, M. L. , Rinaldi, R. , Vanadia, S. , & Colelli, G. (2010). Exposure to 1‐methylcyclopropene (1‐MCP) delays the effects of ethylene on fresh‐cut broccoli raab (*Brassica rapa* L.). Postharvest Biology & Technology, 58(1), 29–35. 10.1016/j.postharvbio.2010.05.001

[fsn3909-bib-0010] Chiellini, E. , & Solaro, R. (2004). Biodegradable polymers and plastics. Chemistry International – Newsmagazine for IUPAC, 26(6), 28–29.

[fsn3909-bib-0011] Cliffebyrnes, V. , & O'Beirne, D. (2005). The effects of cultivar and physiological age on quality and shelf‐life of coleslaw mix packaged in modified atmospheres. International Journal of Food Science & Technology, 40(2), 165–175. 10.1111/j.1365-2621.2004.00927.x

[fsn3909-bib-0012] Darensbourg, D. J. , Ulusoy, M. , Karroonnirum, O. , Poland, R. R. , Reibenspies, J. H. , & Çetinkaya, B. (2009). Highly selective and reactive (salan)CrCl catalyst for the copolymerization and block copolymerization of epoxides with carbon dioxide. Macromolecules, 42(18), 6992–6998. 10.1021/ma901364x

[fsn3909-bib-0013] Del Nobile, M. A. , Licciardello, F. , Scrocco, C. , Muratore, G. , & Zappa, M. (2007). Design of plastic packages for minimally processed fruits. Journal of Food Engineering, 79(1), 217–224. 10.1016/j.jfoodeng.2006.01.062

[fsn3909-bib-0014] Domínguez, I. , Lafuente, M. T. , Hernández‐Muñoz, P. , & Gavara, R. (2016). Influence of modified atmosphere and ethylene levels on quality attributes of fresh tomatoes (*Lycopersicon esculentum* Mill.). Food Chemistry, 209, 211 10.1016/j.foodchem.2016.04.049 27173554

[fsn3909-bib-0015] Dong, T. , Yu, Z. , Wu, J. , Zhao, Z. , Yun, X. , Wang, Y. , … Yang, J. (2015). Thermal and barrier properties of stretched and annealed polylactide films. Polymer Science Series A, 57(6), 738–746. 10.1134/S0965545X15060073

[fsn3909-bib-0016] Dong, T. , Yun, X. , Li, M. , Sun, W. , Duan, Y. , & Jin, Y. (2015). Biodegradable high oxygen barrier membrane for chilled meat packaging. Journal of Applied Polymer Science, 132(16), 41871.

[fsn3909-bib-0017] Dong, T. , Yun, X. , Shi, C. , Sun, W. , Fan, G. , & Jin, Y. (2014). Improved mechanical and barrier properties of PPC multilayer film through interlayer hydrogen bonding interaction. Polymer Science Series A, 56(6), 830–836. 10.1134/S0965545X14060029

[fsn3909-bib-0018] Exama, A. , Arul, J. , Lencki, R. W. , Lee, L. Z. , & Toupin, C. (1994). Suitability of plastic films for modified atmosphere packaging of fruits and vegetables. Journal of Food Science, 58(6), 1365–1370.

[fsn3909-bib-0019] Fernández, K. , Aspe, E. , & Roeckel, M. (2009). Shelf‐life extension on fillets of Atlantic salmon (*Salmo salar*) using natural additives, superchilling and modified atmosphere packaging. Food Control, 20(11), 1036–1042. 10.1016/j.foodcont.2008.12.010

[fsn3909-bib-0020] Fernández‐León, M. F. , Fernández‐León, A. M. , Lozano, M. , Ayuso, M. C. , Amodio, M. L. , Colelli, G. , & González‐Gómez, D. (2013). Retention of quality and functional values of broccoli ‘Parthenon’ stored in modified atmosphere packaging. Food Control, 31(2), 302–313. 10.1016/j.foodcont.2012.10.012

[fsn3909-bib-0021] Flamini, G. , Cioni, P. L. , & Morelli, I. (2003). Differences in the fragrances of pollen, leaves, and floral parts of garland (*Chrysanthemum coronarium*) and composition of the essential oils from flowerheads and leaves. Journal of Agricultural and Food Chemistry, 51(8), 2267 10.1021/jf021050l 12670168

[fsn3909-bib-0022] Fonseca, S. C. , Oliveira, F. A. R. , & Brecht, J. K. (2002). Modelling respiration rate of fresh fruits and vegetables for modified atmosphere packages: A review. Journal of Food Engineering, 52(2), 99–119. 10.1016/S0260-8774(01)00106-6

[fsn3909-bib-0023] Garrido, Y. , Tudela, J. A. , Hernández, J. A. , & Gil, M. I. (2016). Modified atmosphere generated during storage under light conditions is the main factor responsible for the quality changes of baby spinach. Postharvest Biology & Technology, 114, 45–53. 10.1016/j.postharvbio.2015.12.001

[fsn3909-bib-0024] Giuggioli, N. R. , Briano, R. , Baudino, C. , & Peano, C. (2015). Effects of packaging and storage conditions on quality and volatile compounds of raspberry fruits. CyTA – Journal of Food, 13(4), 512–521.

[fsn3909-bib-0025] Harb, J. Y. , & Streif, J. (2004). Controlled atmosphere storage of highbush blueberries cv. ‘Duke’. European Journal of Horticultural Science, 69(2), 66–72.

[fsn3909-bib-0026] Hayakawa, K. I. , Henig, Y. S. , & Gilbert, S. G. (2010). Formulae for predicting gas exchange of fresh produce in polymeric film package. Journal of Food Science, 40(1), 186–191.

[fsn3909-bib-0027] Hernández, J. A. , Rubio, M. , Olmos, E. , Ros‐Barceló, A. , & Martínez‐Gómez, P. (2004). Oxidative stress induced by long‐term plum pox virus infection in peach (*Prunus persica*). Physiologia Plantarum, 122(4), 486–495. 10.1111/j.1399-3054.2004.00431.x

[fsn3909-bib-0028] Herrera, M. A. , Mathew, A. P. , & Oksman, K. (2014). Gas permeability and selectivity of cellulose nanocrystals films (layers) deposited by spin coating. Carbohydrate Polymers, 112(21), 494–501. 10.1016/j.carbpol.2014.06.036 25129773

[fsn3909-bib-0200] Hu, Q. , Fang, Y. , Yang, Y. , Ma, N. , & Zhao, L. (2011). Effect of nanocomposite‐based packaging on postharvest quality of ethylene‐treated kiwifruit (Actinidia deliciosa) during cold storage. Food Research International, 44(6), 1589–1596.

[fsn3909-bib-0029] Hyun, J. E. , & Lee, S. Y. (2017). Effect of modified atmosphere packaging on preserving various types of fresh produce. Journal of Food Safety, 38, e12376.

[fsn3909-bib-0030] Imahori, Y. , Suzuki, Y. , Uemura, K. , Kishioka, I. , Fujiwara, H. , Ueda, Y. , & Chachin, K. (2004). Physiological and quality responses of Chinese chive leaves to low oxygen atmosphere. Postharvest Biology & Technology, 31(3), 295–303. 10.1016/j.postharvbio.2003.09.004

[fsn3909-bib-0031] Irtwange, S. V. (2006). Application of modified atmosphere packaging and related technology in postharvest handling of fresh fruits and vegetables. Agricultural Engineering International: CIGR Ejournal, 8(4), 1946–13.

[fsn3909-bib-0032] Jacxsens, L. , Devlieghere, F. , & Debevere, J. (2002). Predictive modelling for packaging design: Equilibrium modified atmosphere packages of fresh‐cut vegetables subjected to a simulated distribution chain. International Journal of Food Microbiology, 73(2–3), 331 10.1016/S0168-1605(01)00669-9 11934040

[fsn3909-bib-0033] Jamie, P. , & Saltveit, M. E. (2002). Postharvest changes in broccoli and lettuce during storage in argon, helium, and nitrogen atmospheres containing 2% oxygen. Postharvest Biology & Technology, 26(1), 113–116. 10.1016/S0925-5214(02)00006-6

[fsn3909-bib-0034] Jiang, A. L. , Tian, S. P. , & Xu, Y. (2002). Effect of controlled atmospheres with high O_2_ or high‐CO_2_ concentrations on postharvest physiology and storability of “Napoleon” sweet cherry. Journal of Integrative Plant Biology, 44(8), 925–930.

[fsn3909-bib-0035] Kader, A. A. (2008). Flavor quality of fruits and vegetables. Journal of the Science of Food & Agriculture, 88(11), 1863–1868. 10.1002/jsfa.3293

[fsn3909-bib-0036] Kantola, M. , & Helen, H. (2010). Quality changes in organic tomatoes packaged in biodegradable plastic films. Journal of Food Quality, 24(2), 167–176.

[fsn3909-bib-0037] Kasirajan, S. , & Ngouajio, M. (2013). Erratum to: Polyethylene and biodegradable mulches for agricultural applications: A review. Agronomy for Sustainable Development, 33(2), 443 10.1007/s13593-012-0132-7

[fsn3909-bib-0038] Koide, S. , & Shi, J. (2007). Microbial and quality evaluation of green peppers stored in biodegradable film packaging. Food Control, 18(9), 1121–1125. 10.1016/j.foodcont.2006.07.013

[fsn3909-bib-0039] Koning, C. , Wildeson, J. , Parton, R. , Plum, B. , Steeman, P. , & Darensbourg, D. J. (2001). Synthesis and physical characterization of poly(cyclohexane carbonate), synthesized from CO_2_ and cyclohexene oxide. Polymer, 42(9), 3995–4004. 10.1016/S0032-3861(00)00709-6

[fsn3909-bib-0040] Labet, M. , & Thielemans, W. (2009). Synthesis of polycaprolactone: A review. Chemical Society Reviews, 38(12), 3484–3504. 10.1039/b820162p 20449064

[fsn3909-bib-0041] Lee, L. , Arul, J. , Lencki, R. , & Castaigne, F. (2010). A review on modified atmosphere packaging and preservation of fresh fruits and vegetables: Physiological basis and practical aspects—Part I. Packaging Technology and Science, 8(6), 315–331.

[fsn3909-bib-0042] Lee, D. S. , Haggar, P. E. , & Yam, K. L. (1992). Application of ceramic‐filled polymeric films for packaging fresh produce. Packaging Technology and Science, 5(1), 27–30. 10.1002/(ISSN)1099-1522

[fsn3909-bib-0043] Lers, A. , Jiang, W. , Lomaniec, E. , & Aharoni, N. (2008). Gibberellic acid and CO_2_ additive effect in retarding postharvest senescence of parsley. Journal of Food Science, 63(1), 66–68.

[fsn3909-bib-0044] Lidster, P. D. , & Leung, C. K. (2008). Modified atmosphere package systems with gas‐permeable plastic membranes and window for packaging of fresh fruits, vegetables and cut flowers in modified Euro Trays: US.

[fsn3909-bib-0045] Mangaraj, S. , Goswami, T. K. , & Mahajan, P. V. (2009). Applications of plastic films for modified atmosphere packaging of fruits and vegetables: A review. Food Engineering Reviews, 1(2), 133–158. 10.1007/s12393-009-9007-3

[fsn3909-bib-0046] Martínezromero, D. , Bailén, G. , Serrano, M. , Guillén, F. , Valverde, J. M. , Zapata, P. , … Valero, D. (2007). Tools to maintain postharvest fruit and vegetable quality through the inhibition of ethylene action: A review. Critical Reviews in Food Science and Nutrition, 47(6), 543–560. 10.1080/10408390600846390 17653980

[fsn3909-bib-0047] Mistriotis, A. , Briassoulis, D. , Giannoulis, A. , & D'Aquino, S. (2016). Design of biodegradable bio‐based equilibrium modified atmosphere packaging (EMAP) for fresh fruits and vegetables by using micro‐perforated poly‐lactic acid (PLA) films. Postharvest Biology & Technology, 111, 380–389. 10.1016/j.postharvbio.2015.09.022

[fsn3909-bib-0048] Niklis, N. D. , Siomos, A. S. , & Sfakiotakis, E. M. (2002). Ascorbic acid, soluble solids and dry matter content in sweet pepper fruit: Change during ripening. Journal of Vegetable Crop Production, 8(1), 41–51. 10.1300/J068v08n01_06

[fsn3909-bib-0049] Oboh, G. , & Akindahunsi, A. A. (2004). Change in the ascorbic acid, total phenol and antioxidant activity of sun‐dried commonly consumed green leafy vegetables in Nigeria. Nutrition & Health, 18(1), 29 10.1177/026010600401800103 15615324

[fsn3909-bib-0050] Pandrangi, S. , & Laborde, L. F. (2004). Retention of folate, carotenoids, and other quality characteristics in commercially packaged fresh spinach. Journal of Food Science, 69(9), C702–C707.

[fsn3909-bib-0051] Pariasca, J. , Miyazaki, T. , Hisaka, H. , Nakagawa, H. , & Sato, T. (2000). Effect of modified atmosphere packaging (MAP) and controlled atmosphere (CA) on the quality of stored snow pea pods (*Pisum sativum* L. var. saccharatum). Postharvest Biology and Technology, 21, 213–223.

[fsn3909-bib-0052] Qian, M. , Bai, W. D. , Xin, Y. U. , Cai, P. D. , & Xiao, Y. Q. (2009). Effects of CO_2_ on post‐harvest physiology in fruits and vegetables. Science & Technology of Food Industry, 30(10), 350–355.

[fsn3909-bib-0053] Qin, Y. , & Wang, X. (2010). Carbon dioxide‐based copolymers: Environmental benefits of PPC, an industrially viable catalyst. Biotechnology Journal, 5(11), 1164 10.1002/biot.201000134 21058318

[fsn3909-bib-0054] Ranjitha, K. , Rao, D. V. S. , Shivashankara, K. S. , & Roy, T. K. (2015). Effect of pretreatments and modified atmosphere packaging on the shelf life and quality of fresh‐ cut green bell pepper. Journal of Food Science and Technology, 52(12), 7872–7882. 10.1007/s13197-015-1928-7 26604359 PMC4648860

[fsn3909-bib-0055] Sandhya (2010). Modified atmosphere packaging of fresh produce: Current status and future needs. LWT – Food Science and Technology, 43(3), 381–392. 10.1016/j.lwt.2009.05.018

[fsn3909-bib-0056] Severino, R. , Ferrari, G. , Vu, K. D. , Donsì, F. , Salmieri, S. , & Lacroix, M. (2015). Antimicrobial effects of modified chitosan based coating containing nanoemulsion of essential oils, modified atmosphere packaging and gamma irradiation against *Escherichia coli* O157:H7 and *Salmonella typhimurium* on green beans. Food Control, 50, 215–222. 10.1016/j.foodcont.2014.08.029

[fsn3909-bib-0057] Shewfelt, R. L. , Batal, K. M. , & Heaton, E. K. (1984). Broccoli storage: Effect of N6‐benzyladenine, packaging, and icing on color of fresh broccoli. Journal of Food Science, 48(6), 1594–1597.

[fsn3909-bib-0058] Simón, A. , González‐Fandos, E. , & Vázquez, M. (2010). Effect of washing with citric acid and packaging in modified atmosphere on the sensory and microbiological quality of sliced mushrooms (*Agaricus bisporus* L.). Food Control, 21(6), 851–856. 10.1016/j.foodcont.2009.11.012

[fsn3909-bib-0059] Song, S. , Liang, M. , Qi, X. , Jin, Y. , Yang, J. , & Dong, T. (2017). Gas permeability and permselectivity of poly(L‐lactic acid) film by plasma enhanced chemical vapor deposition of SiO_*x*_ . Polymer‐Plastics Technology and Engineering, 82(1), 97–105.

[fsn3909-bib-0060] Song, S. , Liang, M. , Wang, Y. , Liu, L. , Zhang, Y. , Qi, X. , & Dong, T. (2016). Preparation of poly(L‐lactic acid)/SiO_*x*_ film and its gas permeability and permselectivity. Polymer Materials Science & Engineering, 32(11), 135–139.

[fsn3909-bib-0061] Srinivasa, P. , Baskaran, R. , Ramesh, M. , Prashanth, K. H. , & Tharanathan, R. (2002). Storage studies of mango packed using biodegradable chitosan film. European Food Research and Technology, 215(6), 504–508.

[fsn3909-bib-0062] Suparlan, I. K. , (2003). Combined effects of hot water treatment (HWT) and modified atmosphere packaging (MAP) on quality of tomatoes. Packaging Technology and Science, 16(4), 171–178. 10.1002/(ISSN)1099-1522

[fsn3909-bib-0063] Tenorio, M. D. , Villanueva, M. J. , & Sagardoy, M. (2004). Changes in carotenoids and chlorophylls in fresh green asparagus (*Asparagus officinalis* L.) stored under modified atmosphere packaging. Journal of the Science of Food & Agriculture, 84(84), 357–365. 10.1002/(ISSN)1097-0010

[fsn3909-bib-0064] Tharanathan, R. N. (2003). Biodegradable films and composite coatings: Past, present and future. Trends in Food Science & Technology, 54(3), 343–351.

[fsn3909-bib-0065] Tudela, J. A. , Marín, A. , Garrido, Y. , Cantwell, M. , Medina‐Martínez, M. S. , & Gil, M. I. (2013). Off‐odour development in modified atmosphere packaged baby spinach is an unresolved problem. Postharvest Biology & Technology, 75(2), 75–85. 10.1016/j.postharvbio.2012.08.006

[fsn3909-bib-0300] Wang, W. C. , Chiang, Y. A. , Yu, K. J. , Ho, Y. C. , Shen, H. T. , Chang, T. Y. , … Tsao, C. S. (2015). Three‐dimensional digital image correlation measurement of mechanical properties of soft materials[J]. Meccanica, 50(2), 419–428.

[fsn3909-bib-0066] Wang, Y. S. , Tian, S. P. , & Xu, Y. (2005). Effects of high oxygen concentration on pro‐ and anti‐oxidant enzymes in peach fruits during postharvest periods. Food Chemistry, 91(1), 99–104. 10.1016/j.foodchem.2004.05.053

[fsn3909-bib-0067] Watkins, C. B. (2000). Responses of horticultural commodities to high carbon dioxide as related to modified atmosphere packaging. Horttechnology, 10(3), 501–506.

[fsn3909-bib-0068] Xing, Y. , Li, X. , Xu, Q. , Jiang, Y. , Yun, J. , & Li, W. (2010). Effects of chitosan‐based coating and modified atmosphere packaging (MAP) on browning and shelf life of fresh‐cut lotus root (*Nelumbo nucifera* Gaerth). Innovative Food Science & Emerging Technologies, 11(4), 684–689. 10.1016/j.ifset.2010.07.006

[fsn3909-bib-0069] Yun, X. , Wang, Y. , Li, M. , Jin, Y. , Han, Y. , & Dong, T. (2017). Application of permselective poly(ε‐caprolactone) film for equilibrium‐modified atmosphere packaging of strawberry in cold storage. Journal of Food Processing and Preservation, 41, e13247 10.1111/jfpp.13247

[fsn3909-bib-0070] Zorić, Z. , Pedisić, S. , Kovačević, D. B. , Ježek, D. , & Dragović‐Uzelac, V. (2016). Impact of packaging material and storage conditions on polyphenol stability, colour and sensory characteristics of freeze‐dried sour cherry (*Prunus cerasus* var. Marasca). Journal of Food Science and Technology, 53(2), 1247. 27162405 10.1007/s13197-015-2097-4 PMC4837736

